# What is the potential of oligodendrocyte progenitor cells to successfully treat human spinal cord injury?

**DOI:** 10.1186/1471-2377-11-113

**Published:** 2011-09-23

**Authors:** Robert A Watson, Trevor M Yeung

**Affiliations:** 1 Green Templeton College, Woodstock Road, Oxford, OX2 6HG, UK; 2 Stanford University School of Medicine, CCSR 3100, 269 Campus Drive, Stanford, CA 94305, USA

## Abstract

**Background:**

Spinal cord injury is a serious and debilitating condition, affecting millions of people worldwide. Long seen as a permanent injury, recent advances in stem cell research have brought closer the possibility of repairing the spinal cord. One such approach involves injecting oligodendrocyte progenitor cells, derived from human embryonic stem cells, into the injured spinal cord in the hope that they will initiate repair. A phase I clinical trial of this therapy was started in mid 2010 and is currently underway.

**Discussion:**

The theory underlying this approach is that these myelinating progenitors will phenotypically replace myelin lost during injury whilst helping to promote a repair environment in the lesion. However, the importance of demyelination in the pathogenesis of human spinal cord injury is a contentious issue and a body of literature suggests that it is only a minor factor in the overall injury process.

**Summary:**

This review examines the validity of the theory underpinning the on-going clinical trial as well as analysing published data from animal models and finally discussing issues surrounding safety and purity in order to assess the potential of this approach to successfully treat acute human spinal cord injury.

## Background

Clinicians currently have very limited treatment options when managing a patient with spinal cord injury (SCI). Symptom control, supportive care and rehabilitation are the mainstays of treatment [1] and a reversal of the injury process has, until recently, been science fiction. However, developments in the field of stem cells have led many to believe that regeneration of an injured spinal cord is indeed possible and one such approach is currently undergoing a phase I clinical trial [2]. In the first trial of its kind, oligodendrocyte progenitor cells (OPCs) derived from human embryonic stem cells (hESCs) will be injected into the spinal cords of patients with an acute thoracic SCI to assess safety and efficacy in restoring neurological function [3]. Initially hailed as a major advancement [4] and treated with optimism in the popular press [5], this trial has already experienced setbacks and was placed on clinical hold by the FDA in 2009 due to safety issues [6]. Whilst the trial has now been given permission to proceed [7], lessons learnt from French gene therapy trials in 2003, in which retroviral gene insertion triggered leukaemia in some patients, show that any failures can potentially set back an entire field [8]. If similar serious issues of safety or efficacy arise, the effect on investors, regulators and patients may be extremely damaging.

### Pathogenesis of SCI

#### Epidemiology

SCI, caused by accidental injury in most cases [9], has universal importance but is particularly relevant to young people, with the average injury age 33 years [9]. A moment of trauma sets in motion a disease process ultimately resulting in partial or full paralysis and loss of some or all sensory input below the injury level, clinically known as incomplete or complete injury respectively [10]. With no cure currently available, patients can expect to spend the rest of their life in a wheelchair and approximately 16% of patients (those with complete tetraplegia [11]) face the prospect of life-long full paralysis from the neck down. In addition, many of the millions of patients worldwide also experience incontinence [12], chronic pain [13] and psychiatric disorders [14,15]. This has an important social toll as many patients who have suffered an SCI experience significant disruption to their social and work lives, having a profound impact on their family and society around them [16]. Further, the direct costs of caring for patients with an SCI are enormous and are estimated to be in the region of $7 billion per annum in the US [17].

#### Primary and secondary disease

The pathophysiology of SCI is biphasic, comprising a primary and secondary injury phase [18]. The primary injury phase refers to the injury itself, consisting of mechanical disruption of tissue caused by the force imparted by the primary injury mechanism. Although every human injury is unique, the most common injury mechanism is contusion (analogous to soft tissue bruising), often with prolonged compression due to crushing of the vertebrae [19]. Hyper-bending, hyper-stretching, rotation and laceration can also occur [10], although complete transections of the cord are rare and usually intact white matter is seen to traverse the lesion [20,21]. Whilst tissue damage can occur during this immediate phase, there is a surprising paucity of permanent pathological changes, underscoring the importance of secondary injury mechanisms [22].

**Figure 1:**
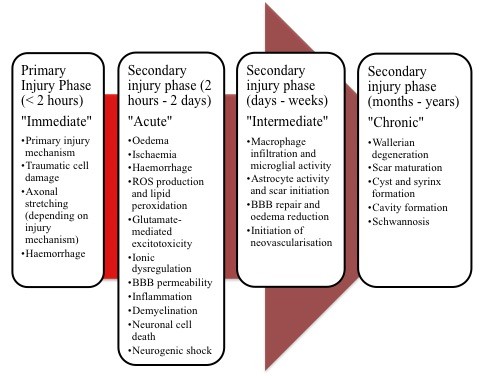
**The key events following SCI**. Adapted from [19-22]. BBB: Blood Brain Barrier; ROS: Reactive Oxygen Species.

The secondary injury phase depends on the time post injury and the processes occurring (summarised in Figure 1). Most damage and cellular loss occurs during the acute and intermediate phases where an extensive range of processes result in widespread apoptosis and necrosis of both neurons and oligodendrocytes, leading to neurological deficits [22-24]. The late intermediate and chronic phases are characterised by progressive degeneration, accompanied by attempts at endogenous repair [19]. Morphological changes associated with the chronic phase (Figure 1) are significant barriers to any cell replacement therapy and hence the clinical protocol for the use of OPCs involves administration during the intermediate phase, 7-14 days post injury [25,26].

### Stem cell therapies and SCI

It is possible to classify 'stem cells' into two broad categories, embryonic and somatic; the latter term including all stem cells present in the adult as well as those found in foetal and neonatal tissues [27]. Embryonic stem cells are derived from the human blastocyst and maintain the ability to differentiate into any cell type (pluripotency) [28], whilst somatic stem cells are able to differentiate into a limited number of cell lineages (multipotency). It is this ability to differentiate into a variety of cell types, as well as secrete growth factors, that has gained interest from the field of spinal cord injury. It is hoped that stem cells can be used to replace neural cells lost during the injury process and stimulate a repair environment [29]. A number of stem cell types including mesenchymal stem cells, olfactory ensheathing cells and neural stem cells have been considered for use in SCI. However, further discussion of the use of these cell types is outside the scope of this article and is well reviewed elsewhere [27,29-33].

The cell type currently being trialled in human patients, and the focus of this article, is the oligodendrocyte progenitor cell. These cells are derived from human embryonic stem cells by culturing them in conditions to drive commitment to an oligodendroglial lineage [34,35]. Once purified, up to 95% of these cells display markers characteristic of oligodendroglial cells - GalC, RIP and O4 - and morphologically resemble oligodendroglial cells [26]. These progenitors can then be injected into the spinal cord where, in a demyelinating environment, they further differentiate into oligodendrocytes - the myelinating cells of the central nervous system [26]. In the human SCI lesion site, it is hoped that OPCs will work as a "combination therapy" [29] - phenotypically replacing lost oligodendrocytes and hence remyelinating axons that have become demyelinated during SCI, as well as secreting neurotrophic factors to establish a repair environment in the lesion [29].

This review sets out to assess the potential of this treatment to successfully heal SCI by first looking at the role of demyelination in the pathogenesis of SCI. We then assess the importance of trophic support and the repair environment before analysing *in vivo *models of OPC treatment. Finally, practical, safety and ethical issues will be discussed.

## Discussion

### The role of demyelination and axonal sparing in SCI

One of the key premises of using OPC transplants for treatment of SCI is that demyelination is a major contributor to the pathogenesis of the injury. Clearly, in order for remyelinating therapies to succeed there must be appropriate targets - i.e. naked, intact axons - in which function is restored to some degree following treatment. Somewhat surprisingly, the existence of such targets following human SCI is controversial and casts doubt on much of the fundamental science underlying OPC treatment. Whilst a number of animal studies [36,37] have demonstrated myelin loss with sparing of the associated axon - so-called 'primary demyelination' - the extent to which this occurs following human injury is ambiguous and a number of post mortem studies of human SCI have suggested that it may not occur to a significant degree [20,22,38].

It may be possible to explain the observed difference in the extent of primary demyelination between *post mortem *human samples and animal models. Experimental data demonstrates that axons need myelin for trophic support - mice missing two major myelin proteolipids, PLP and DM-20, typically show axonal swelling and degeneration [39]. Therefore any naked axons in humans may degenerate if they are not provided with support from oligodendrocytes and hence wouldn't be detected *post mortem*. Additional mouse data has supported this theory by showing a close correlation between myelin status and axonal survival [40].

Nonetheless, there are a number of inconsistencies with this explanation. Firstly, investigations in rats have identified chronically demyelinated axons [37], suggesting that some axons are capable of surviving without oligodendrocyte support. Additionally, were this explanation valid, then one would expect to see naked axons in acute or intermediate stage *post mortem *lesions, before they had time to degenerate. However, this is not reported to be the case [20,22,38].

In summary, the role of demyelination is far from clear. Whilst some animal studies [36,37] suggest that naked, primary demyelinated axons - the target for OPC treatment - exist, *post mortem *human studies do not corroborate this [20,22,38]. This disparity could be due to the explanation given above, however, as discussed, this has a number of caveats. Nonetheless there could be other reasons for the conflicting results between human and animal studies such as the fact that *post mortem *samples - particularly from acute or intermediate phase lesions - necessarily come from injuries associated with high mortality and hence represent a more severe lesion. As suggested by Guest *et al *(2005), primary demyelination is more likely in less severe injury where axons can survive but oligodendrocytes cannot [38]. Perhaps, as suggested by Rowland et al, emerging magnetic resonance imaging technologies such as magnetization transfer and diffusion tensor imaging which allow the structural integrity of tissue to be imaged in live patients will clarify the situation in humans [19].

Overall, despite the prominent role for demyelination suggested in animal studies, a lack of consensus on the importance of this process in humans creates uncertainty regarding how successfully OPCs can treat human SCI. Indeed, even if remyelination is a successful strategy, there are many barriers to clinical improvement. For example, glial scar formation can create a physical barrier to OPCs whilst a number of molecules can have an inhibitory effect on OPCs. For example, both chondroitin sulphate proteoglycans (CSPGs) and TNF-α found within the scar have been shown to reduce OPC growth [41,42], whilst other studies have shown that bone morphogenic protein (BMP) produced by astrocytes can reduce OPC differentiation into oligodendrocytes and promote differentiation into astroctyes [43].

### Trophic support and the importance of a repair environment

OPC transplantation is a dual strategy, aiming not just to allow remyelination but also providing trophic support and a repair environment [44]. Spinal cord injury sets in motion a plethora of repair mechanisms, including endogenous remyelination and increased expression of a number of neurotrophic factors such as transforming growth factor-β2 (TGF-β2) and brain-derived neurotrophic factor (BDNF) [45,46]. Such up-regulation is thought to contribute to neuroprotection and even axonal sprouting [47]. However, axonal sprouting and endogenous repair is often abortive - due, in part, to an insufficient repair environment to overcome inhibitory mechanisms or promote maintained regeneration [48]. Moreover, although endogenous remyelination is often seen following SCI, it is usually functionally and anatomically incomplete [48-50]. It is therefore hoped that the grafting of a large number of OPCs soon after injury will create a 'repair environment' and allow these processes to develop further.

A host of evidence is weighing up in favour of this theory - OPCs are capable of producing numerous neurotrophins including midkine, TGF-β2 and BDNF, contributing to a repair environment [51] and even promoting neurite outgrowth of rat sensory neurons *in vitro *[51]. *In vivo *studies (discussed in more detail below) have also suggested that OPC transplantation can significantly alter lesion pathogenesis and influence gene expression towards an uninjured pattern [52]. Indeed, in one experimental system, exposure of media conditioned by OPCs and oligodendrocytes alone was enough to increase the survival and axonal lengthening of neurons [53]. Therefore it is possible that even in the absence of remyelination, OPC transplantation may bring clinical improvement by providing trophic support and creating a regenerative environment, preventing the cellular damage and apoptosis seen in the secondary injury phase.

Despite this, eliciting neural regrowth is an ambitious goal as despite early optimism, neurotrophin based therapies, which aim to induce a repair environment, have been largely unsuccessful [54-56] and harnessing this process has remained elusive. Moreover, sprouting can also be pathogenic as new connections made by sensory afferents can lead to neuropathic pain [57] and autonomic dysreflexia [58]. Further, the inflammatory milieu following SCI is extremely complex and so the timing of any acute graft needs to be extremely carefully considered [59]. Hence whilst attempting to provide a supportive environment is a reasonable approach, it may be difficult to achieve in practice.

In summary, the potential benefit of remyelination remains controversial due to disagreements in the role of this process in human SCI. In addition, even if a remyelinating strategy is the correct one, there are a number barriers to success remaining including scar formation, inhibitory molecules and the complexity of the inflamed lesion. The latter also makes generation of a repair environment somewhat complex. These factors leave challenges to be overcome in the development of this therapy.

### In vivo evidence for potential of OPC transplantation

Despite much debate surrounding the underlying principles of the potential of OPC transplants, a number of whole body *in vivo *experiments have been conducted to assess OPC-based therapy. The most important of these in the public domain, cited on the announcement of the clinical trial [4], is that of Keirstead et al (2005) [44]. The authors successfully demonstrated that injection of OPCs into rats with a thoracic contusion SCI seven days post-injury led to remyelination and restoration of some locomotor function. A similar study into cervical SCI [52], conducted by the same group, obtained similar results - demonstrating that OPC transplantation can improve forelimb motor function as well as altering lesion pathogenesis; shown by increased white and grey matter sparing, decreased cavitation and altered gene expression [52]. This, too, was claimed to be "proof-of-concept" for the OPC clinical trials in a separate press release [60]. Subsequently, other groups have also shown neurological improvements following OPC injection into rodent models of SCI, further validating this approach [61-63]. Whilst these papers [44,52] undoubtedly offer strong evidence for the promise of OPC therapy, there are a number of considerations to be made before these results can be applied to human therapy.

Firstly, contusion injury was produced by an 'impactor' - a device which transiently delivers a specific force to the posterior of the spinal cord [64]. It is questionable how accurately this models human SCI where a variety of injury mechanisms often occur anteriorly [65], followed by prolonged compression, spinal fractures [66], haemorrhage and inflammation [67] and serious systemic injuries. Secondly, all SCIs were elicited under anaesthesia, followed by sterile wound closure and antibiotics [44,52]. Anaesthesia is known to have neuroprotective properties [68,69], and may therefore alter the injury outcome, whilst antibiotics prevent infection, a factor that can exacerbate human SCI [9]. Thirdly, all animals were surgically prepared before injury - the paravertebral muscles were dissected and the spinal laminae removed. This procedure is known to reduce secondary disease processes such as swelling and oedema, altering disease pathogenesis [70]. Furthermore, these studies used locomotor function as a guide for neurological improvement but made no attempt to examine sensory or autonomic function - something that any treatment of SCI needs to address. Additionally, in the 2005 study, locomotion was assessed, in part, using the Basso Beattie and Bresnahan (BBB) scale [44], the sensitivity and reproducibility of which has been criticised [71]. Finally it is of interest to note that the authors of the 2005 study declare "no potential conflict of interest" [52] despite their work being funded by the company running the clinical trial, and being named by the latter as 'collaborators' [60]. This is probably of no consequence, but increased transparency would likely bestow greater confidence in the results obtained.

Whilst it is possible to criticise the extrapolation of animal data to humans, particularly in the context of SCI [72], one must bear in mind that whilst small-animal models may be flawed, they are still an important tool and live *in vivo *experiments are a crucial element of preclinical investigation. In spite of the aforementioned conflict of interest, the studies were rigorously conducted with appropriate controls and blinding of investigators where necessary. The fact that significant positive results were obtained, for both thoracic and cervical injury, provides a reasonable basis for development of this treatment. Whilst historically, promising animal data has often translated poorly into clinical success [72], it remains to be seen whether this will be true in this case.

### Practical issues

#### Safety and purity

Transplanting pluripotent cells derived from embryos into adult humans clearly has associated risks. Two major possible adverse effects are teratoma formation and allogeneic rejection but a number of other complications could also potentially arise. Issues surrounding the safety and purity of the OPCs need to be carefully considered, as they could be a major barrier to the success of this therapy.

#### Teratoma formation

Human embryonic stem cells (hESCs) share a number of phenotypes with tumour cells - for example a rapid replication rate, genetic instability and telomerase activity [73]. On injection into immunodeficient mice, hESCs inevitably form teratomas (benign tumours comprising of tissue from all three embryonic layers) and indeed this association is so strong that this has become a test of pluripotency [73]. To mitigate the risk of teratoma formation, it is possible to employ one of three strategies: differentiation of pluripotent stem cells, manipulation of genetics to prevent tumorigenesis or careful surveillance and removal of any tumours that form in the patient [74].

**Figure 2:**
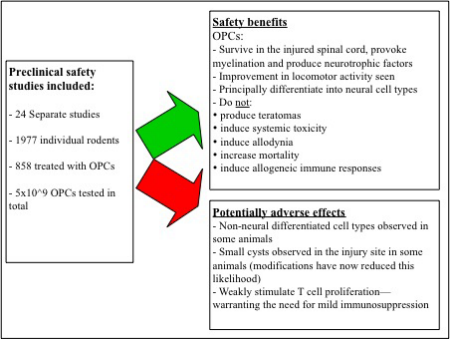
**Conclusions from non-clinical safety studies of the OPCs to be injected into human spinal cords**. Adapted from [25].

Therapies involving OPCs are applying the first of these strategies by pre-differentiating hESCs into progenitor cells, abrogating the risk of teratoma formation [26]. Throughout intensive preclinical screening no evidence of aberrant growth or teratomas was found [75], implying that this strategy was successful. However, the risk of tumour formation a number of years after grafting, or due to contamination with undifferentiated hESCs, cannot be eliminated, especially as it is extremely difficult to obtain OPCs with 100% purity [34,44]. New culture techniques may improve purity (discussed below) but the risk of contamination remains valid. Indeed, during animal studies, non-neural tissue and cysts were found in some cases (Figure 2) and the current trial was placed on clinical hold for over a year pending further investigation by the FDA [6]. Whilst the trial has now resumed, it is not possible to predict the long-term consequences and risks at this stage.

#### Immunogenicity and xenogenic culture

**Figure 3:**
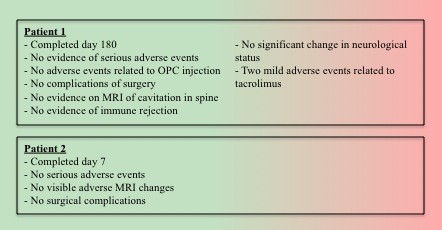
**Outcomes reported in June 2011 of the phase I clinical trial of OPCs in human lesions**. Adapted from [91]

Studies evaluating the immunogenicity of OPCs indicate that they elicit only a mild immune response [76], perhaps a product of the low immunogenicity of hESCs [77,78], although some authors have suggested that this may not always be the case [79]. Either way, OPCs are an allogeneic transplant and as such pose a risk of immune rejection and subsequent exacerbation of any lesion. For this reason, the trial protocol includes low-dose immunosuppression [2,25]. However, immunosuppressive therapy is known to have a number of side effects and in fact the first patient in the current phase I trial experienced a "mild" adverse event relating to tacrolimus (Figure 3).

The propensity to cause immune rejection or tumour formation is increased if the sample of cells grafted is impure. Many differentiation protocols, including the one used for the on-going trial [80], still use xenogenic products which can lead to alterations in cell surface molecules and carries a risk of pathogen cross-transfer. Recent advances have led to the emergence of xeno-free culturing protocols [81] which may facilitate the derivation of purer samples of donor cells and hence may help to mitigate the chance of adverse events.

Overall, risk analysis and preclinical testing for OPCs has been thorough and many other potential side effects such as allodynia or adverse effects on locomotion have been extensively screened for (Figure 2) and found to be of minimal risk [25,82]. However, it is not possible to ever eliminate the risk of adverse events and due to the variety and capriciousness of side effects, combined with cautious regulation, safety concerns could prove a serious hindrance to OPC treatment.

#### Commercial viability

OPCs are commercially viable due to the fact that they are durable, capable of tolerating freeze-thaw cycles and immortal [29]. This makes laboratory handling straightforward and standardisation and scaling up of manufacturing possible. Commercial viability is a very important factor for bringing new treatments to the clinic and the fact that OPCs fulfil these criteria enhances their potential to succeed. However, were OPCs to suffer a major setback during their first trial, investments - currently sustaining the on-going trial - may start to wane, thus jeopardising any future potential success.

#### Ethical issues

Due to complex ethical issues [83], there is a corpus of opposition to hESC research and associated therapies [84]. These ethical dilemmas are diverse, but in summary, opposition tends to focus on the fact that research using human embryos violates the sanctity of life, with necessary destruction of these embryos tantamount to killing a human and hence morally unjustifiable [85]. Proponents of hESC research would counter that the moral status of a blastocyst is ambiguous and that whilst destruction of an embryo is undesirable, it is vindicated by the benefits it brings [85]. Furthermore, many embryos are 'spares' from IVF clinics, destined for destruction [85]. This is a simplistic overview, but regardless of any debate, opposition to this work is likely to always remain. Although private corporations will probably always be able to continue with hESC research, especially in an international context, any failures in this field would do little to quell opposition, potentially slowing future progress.

Recently, ethical concerns have also been raised regarding the on-going clinical trial. In particular, Bretzner *et al *(2011) raised objections to the design and patient population [86]. They claim that patients diagnosed with an acute SCI may be especially vulnerable to "therapeutic misconception", whereby they believe and are motivated by the idea that they will gain therapeutic benefit from the trial [86]. Further, they suggest that enrolling patients in the acute phase risks jeopardizing any spontaneous recovery and that there are more appropriate patient populations for a phase I safety trial of OPCs [86]. Whilst those responsible for the trial have strongly defended the chosen protocol [87] and other commentators have recently written critiques of Bretzner *et al*'s article [88], it is clear that this pioneering study is, and will continue to be, subject to intense scrutiny and opposition. This pressure is likely to augment the consequences of any success or failure.

#### Progress of the clinical trial

The on-going phase I clinical trial into the use of OPCs for spinal cord injury has now been in progress for 9 months. Two patients have so-far undergone treatment and updates on the trial have recently been given at conferences in the first half of 2011 [89]. The trial is continuing to recruit patients [89]. The investigators state that "no serious safety issues have occurred to date" [90] and whilst no conclusions can be drawn until the trial has reached completion and the data appropriately collected and analysed, it is promising that there have been no major setbacks since its commencement (Figure 3) [91]. It should also be noted, however, that as this is a phase I clinical trial, the primary outcome is safety. Hence it will not be until the completion of phase II and III efficacy trials that inferences about the effectiveness of this therapy can be confidently drawn.

## Summary

This review set out to assess the potential of OPCs to treat SCI by examining injury pathogenesis, current data from whole-body *in vivo *models and practical considerations. The situation surrounding pathogenesis is unclear, with opinion regarding the role of demyelination in human injury divided. Animal data - albeit from researchers keen to develop new technologies - suggests an important role for demyelination whilst clinical samples, notwithstanding their own caveats, indicate the contrary. Resolution of this issue may only come with new technologies or indeed the outcome of the planned trial and it is possible that OPCs will have clinically beneficial effects on the course of SCI independent of remyelination by providing trophic support in the injured spine.

Whole-body models have been promising, although extrapolation of rodent data to complicated human injury is questionable and history warns caution in this regard. There are numerous safety and purity issues regarding the culture mechanisms and potential tumorigenic nature of OPCs and any problems may lead to major setbacks for the field. Finally whilst the investment and ethical climate is currently favourable, turbulence in either of these facets could hinder future treatments.

In summary, OPCs have the potential to treat SCI, but, as discussed in this review, there are many hurdles to overcome and numerous uncertainties. So far, animal studies have shown that any observable improvements are modest and that OPC transplants are only successful in acute lesions [44], so chronically injured patients or those expecting miracles risk disappointment. Nonetheless, SCI is a field in need of progress, exemplified by the fact that many patients are so desperate for a cure that they are driven abroad for risky and expensive procedures [92-94]. Only time will tell if OPCs will treat SCI, but whilst they have potential, there is at least hope for success.

## Competing interests

The authors declare that they have no competing interests.

## Authors' contributions

RAW elaborated the general structure of the manuscript and drafted it. TMY critically revised the draft. All authors have read and approved the final version of the manuscript.

## Pre-publication history

The pre-publication history for this paper can be accessed here:

http://www.biomedcentral.com/1471-2377/11/113/prepub
